# Elucidating the Phytochemical Landscape of Leaves, Stems, and Tubers of *Codonopsis convolvulacea* through Integrated Metabolomics

**DOI:** 10.3390/molecules29133193

**Published:** 2024-07-04

**Authors:** Fang Yuan, Shiying Yan, Jian Zhao

**Affiliations:** 1 Key Laboratory of Biological Resource and Ecological Environment of Chinese Education Ministry, College of Life Sciences, Sichuan University, Chengdu 610065, China; yfsamantha-06@163.com (F.Y.); 13107178792@163.com (S.Y.); 2 Key Laboratory of Tibetan Medicine Resources Conservation and Utilization of Tibet Autonomous Region, Tibet Agriculture and Animal Husbandry University, Nyingchi 860000, China

**Keywords:** *Codonopsis convolvulacea*, medicinal plants, volatiles, terpenoids, phytochemicals

## Abstract

*Codonopsis convolvulacea* is a highly valued Chinese medicinal plant containing diverse bioactive compounds. While roots/tubers have been the main medicinal parts used in practice, leaves and stems may also harbor valuable phytochemicals. However, research comparing volatiles across tissues is lacking. This study performed metabolomic profiling of leaves, stems, and tubers of *C. convolvulacea* to elucidate tissue-specific accumulation patterns of volatile metabolites. Ultra-high performance liquid chromatography–tandem mass spectrometry identified 302 compounds, belonging to 14 classes. Multivariate analysis clearly differentiated the metabolic profiles of the three tissues. Numerous differentially accumulated metabolites (DAMs) were detected, especially terpenoids and esters. The leaves contained more terpenoids, ester, and alcohol. The stems accumulated higher levels of terpenoids, heterocyclics, and alkaloids with pharmaceutical potential. The tubers were enriched with carbohydrates like sugars and starch, befitting their storage role, but still retained reasonable amounts of valuable volatiles. The characterization of tissue-specific metabolic signatures provides a foundation for the selective utilization of *C. convolvulacea* parts. Key metabolites identified include niacinamide, p-cymene, tridecanal, benzeneacetic acid, benzene, and carveol. Leaves, stems, and tubers could be targeted for antioxidants, drug development, and tonics/nutraceuticals, respectively. The metabolomic insights can also guide breeding strategies to enhance the bioactive compound content in specific tissues. This study demonstrates the value of tissue-specific metabolite profiling for informing the phytochemical exploitation and genetic improvement of medicinal plants.

## 1. Introduction

*Codonopsis convolvulacea*, commonly known as “dang shen”, is a highly valued traditional Chinese medicinal plant that has been used for centuries in China [1]. The species is widely distributed in southern provinces, including Sichuan, Yunnan, and Guizhou. It thrives in shady and humid mountainous areas [2]. The genus Codonopsis, which belongs to the family Campanulaceae, contains more than 40 species that are mainly distributed in Asia, of which about 39 species have been found in China [3,4]. These species are integral to traditional Tibetan medicine and are recognized within the Chinese pharmacopeia. Among the various species, *C. pilosula* is particularly well-known. As one of the 50 fundamental herbs in Chinese medicine, *C. convolvulacea* features extensively in herbal formulations, as a tonic adaptogen and a treatment for qi, blood, yin, and yang deficiencies [2,5].

Triterpenoid saponins, polysaccharides, alkaloids, and polyphenols have been identified as the major bioactive components in *C. convolvulacea* [3,6]. These compounds have shown diverse pharmacological activities, including immunostimulating, anti-inflammatory, antioxidative, anticarcinogenic, neuroprotective, and cardioprotective activities [7,8]. In traditional practice, C. convolvulacea is commonly prescribed to enhance qi, protect cardiovascular health, reduce blood glucose, improve memory, and boost immune function. It is also used to treat respiratory and digestive complaints. Owing to its medicinal significance, *C. convolvulacea* has been cultivated across China for centuries [2]. Both wild and cultivated varieties are employed, although cultivated strains typically achieve higher yields and saponin content. Propagation is via seeds, cuttings, or tissue culture. Ideal growing conditions are shady, moist mountain habitats, with well-drained sandy loam soils. Mature roots and rhizomes are harvested at 3-4 years, for medicinal use.

The major bioactive components identified in *C. convolvulacea* are triterpenoid saponins, polysaccharides, alkaloids, and polyphenols [3]. These compounds have exhibited diverse pharmacological activities, including immunostimulatory, anti-inflammatory, antioxidant, anticancer, neuroprotective, and cardioprotective effects [7,8,9,10]. Key triterpenoid saponins, such as lobetyolinin, convulvosides, and integripetalosides, are credited with many of these bioactivities [9].

While roots and rhizomes have been the dominant medicinal parts used in practice, *C. convolvulacea* leaves, stems, and seeds may also harbor bioactive chemicals [3]. However, research on the explicit profiling and comparison of volatile metabolites in these different tissues is scarce. Most prior chemical analyses have focused solely on roots/tubers or whole plants. Detailed metabolomic studies evaluating the three key tissues, namely stems, leaves, and tubers, are lacking.

Metabolomic techniques enable comprehensive profiling of the diverse, small molecule metabolites produced by an organism [11,12]. With increased research efforts to discover novel bioactive compounds, the field of plant metabolomics is rapidly expanding [13]. These studies are important to detect adulteration, to evaluate the relationship between metabolite concentrations and biological activities, and to ensure quality control of medicinal plants [14,15]. This burgeoning area of study is central to advancing our understanding of plant biochemistry and enhancing the therapeutic efficacy of plant-derived medicines. Metabolic profiling of medicinal plants like *C. convolvulacea* can provide insights into their phytochemical reservoirs. Characterizing tissue-specific metabolite distribution using modern metabolomic tools can inform better utilization of different plant parts.

The objectives of this study were to use metabolomic techniques to comprehensively profile and contrast the chemical composition of *C. convolvulacea* stems, leaves, and tubers. The goals were to elucidate the distribution of volatiles among these tissues and gain insights into their traditional medicinal applications and potential modern pharmacological uses. This tissue-specific metabolite mapping will also inform future breeding, cultivation, and processing methods to optimize the medicinal value of this important Chinese herb.

## 2. Results 

### 2.1. Antioxidant Activity

**Figure 1:**
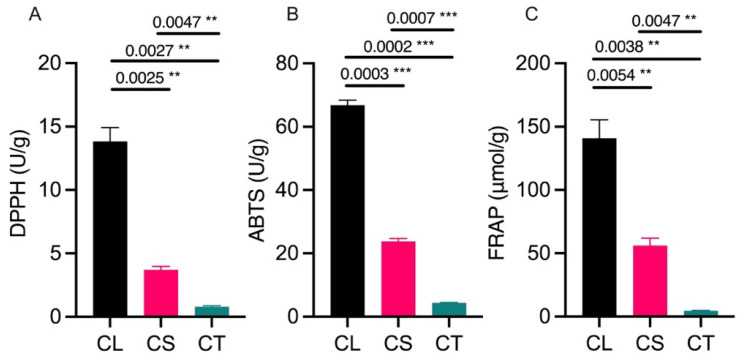
Antioxidant activity estimated through multiple assays: (**A**) DDPH (2,2-diphenyl-1-picrylhydrazyl) assay, (**B**) ABTS (2,2-azinobis (3-ethyl-benzothiazoline-6-sulfonic acid) assay, and (**C**) FRAP (ferric reducing antioxidant power) assay. CL = leaf tissues, CS = stem tissues, CT = tuber tissues, ** = significant at *p* < 0.01, and *** = significant at *p* < 0.001.

The antioxidant activity was assessed using three methods, namely a DPPH radical scavenging assay, an ABTS radical cation decolorization assay, and a ferric reducing antioxidant power (FRAP) assay (Figure 1). The leaf samples showed the highest antioxidant activity in all three assays, with average values of 13.818 U/g for the DPPH assay, 66.836 U/g for the ABTS assay, and 140.859 μmol/g for the FRAP assay. The antioxidant activity was lowest in the tuber samples, which averaged 0.799 U/g, 4.356 U/g, and 4.541 μmol/g for the DPPH, ABTS, and FRAP assay, respectively (Figure 1A–C). The stems had intermediate antioxidant activity, with mean values of 3.711 U/g, 23.779 U/g, and 56.101 μmol/g for the DPPH, ABTS, and FRAP assay. The high antioxidant activity in the leaves can be attributed to the abundance of phenolic antioxidants, such as flavonoids.

### 2.2. Proximate Composition

**Figure 2:**
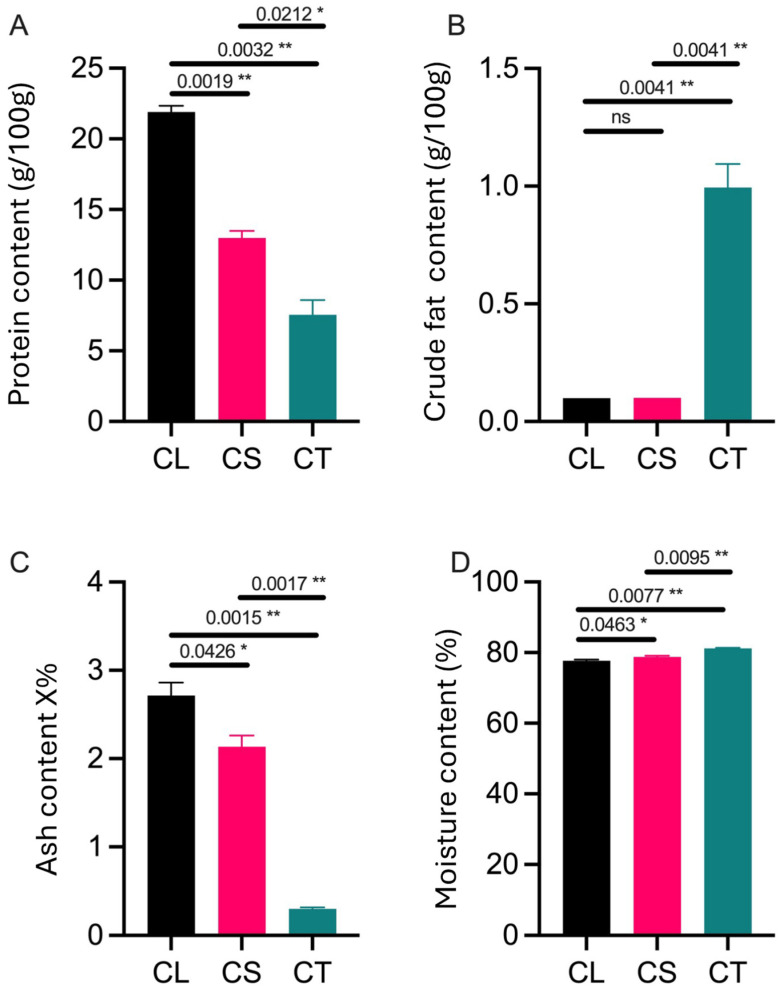
Proximate composition analysis of leaf, stem, and tuber tissues: (**A**) estimation of protein content, (**B**) estimation of crude fat content, (**C**) estimation of ash content, and (**D**) estimation of moisture percentage. CL = leaf tissues, CS = stem tissues, CT = tuber tissues, * = significant at *p* < 0.05, and ** = significant at *p* < 0.01.

The proximate analysis showed some apparent differences in nutrient composition across the three tissues (Figure 2). The leaves had the highest protein content, averaging 21.904 g/100 g, followed by the stems (12.990 g/100 g), and tubers (7.554 g/100 g) (Figure 2A). The fat content was very low in all the tissues, ranging from 0.099–0.100 g/100 g in the leaves and stems, to 0.995 g/100 g in the tubers (Figure 2B). The ash content was also relatively low and comparable in the leaves (2.712%) and stems (2.134%), but slightly lower in the tubers (0.301%) (Figure 2C). As expected, the tubers had the highest moisture content, averaging 81.191%, compared to 77.672% in the leaves and 78.830% in the stems (Figure 2D).

### 2.3. Total Amino Acids

**Figure 3:**
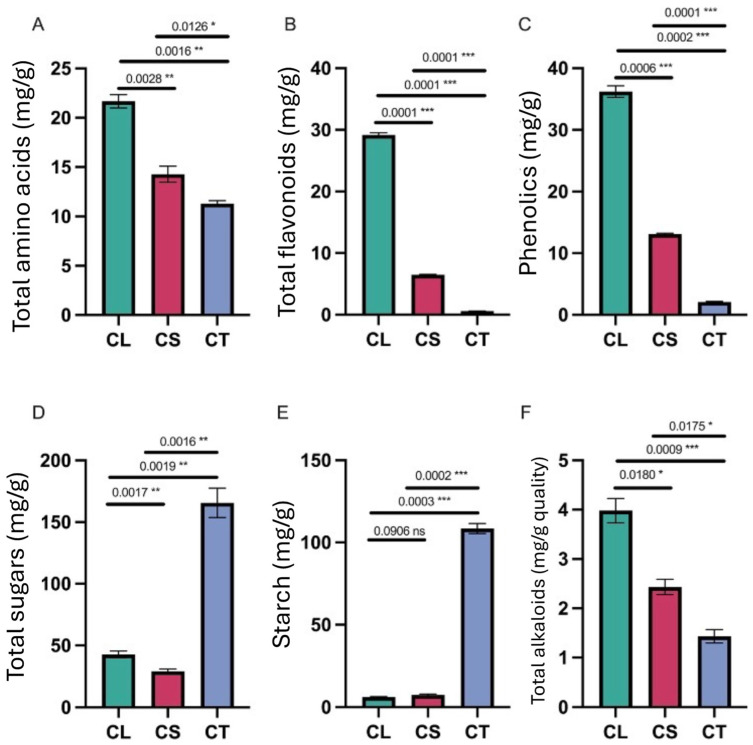
Phytochemical analysis of leaf, stem, and tuber tissues: (**A**) total amino acids, (**B**) total flavonoids, (**C**) phenolics, (**D**) total sugars, (**E**) starch content, and (**F**) total alkaloids. CL = leaf tissues, CS = stem tissues, CT = tuber tissues, * = significant at *p* < 0.05, ** = significant at *p* < 0.01, and *** = significant at *p* < 0.001.

The measurement of the total amino acids (Figure 3A) revealed the highest level in the leaves (21.672 μmol/g), followed by the stems (14.286 μmol/g), and then the tubers (11.295 μmol/g). This aligns with the trend observed for the crude protein content. The abundance of amino acids and protein in the leaves can be attributed to active photosynthesis.

### 2.4. Total Flavonoids and Phenolics

The total flavonoid content (Figure 3B) was markedly higher in the leaves versus the other tissues. The leaves contained 29.514 mg/g total flavonoids on average, while the stems had 6.481 mg/g, and the tubers just 0.573 mg/g. Similarly, the leaves contained the highest level of total phenolics at 36.212 mg/g, compared to 13.103 mg/g in the stems and 2.074 mg/g in the tubers (Figure 3C). These results correlate well with the strong antioxidant activity exhibited by the leaf samples.

### 2.5. Total Sugars and Starch

The tubers contained significantly higher levels of sugars and starch compared to the leaves and stems (Figure 3D,E). The average total sugar content was 165.654 mg/g in the tubers, 32.405 mg/g in the leaves, and 28.681 mg/g in the stems. Starch level was 108.519 mg/g in the tubers, 6.542 mg/g in the leaves, and 7.349 mg/g in the stems. This large accumulation of carbohydrates highlights the role of tubers as storage organs.

### 2.6. Total Alkaloids

The alkaloid content (Figure 3F) was highest in the leaves (3.981 mg/g), followed by the stems (2.432 mg/g), and then the tubers (1.432 mg/g). Many medicinally active compounds in *C. convolvulacea* belong to the alkaloid class. The abundance of alkaloids in the leaves suggests potential pharmacological uses.

In brief, the metabolite profiling indicates that leaves contain high levels of antioxidants, amino acids, flavonoids, phenolics, and alkaloids. The stems also contain useful levels of these compounds, while the tubers are rich in carbohydrates and moisture, but poorer in bioactive metabolites.

### 2.7. Overview of Metabolic Profiling

**Figure 4:**
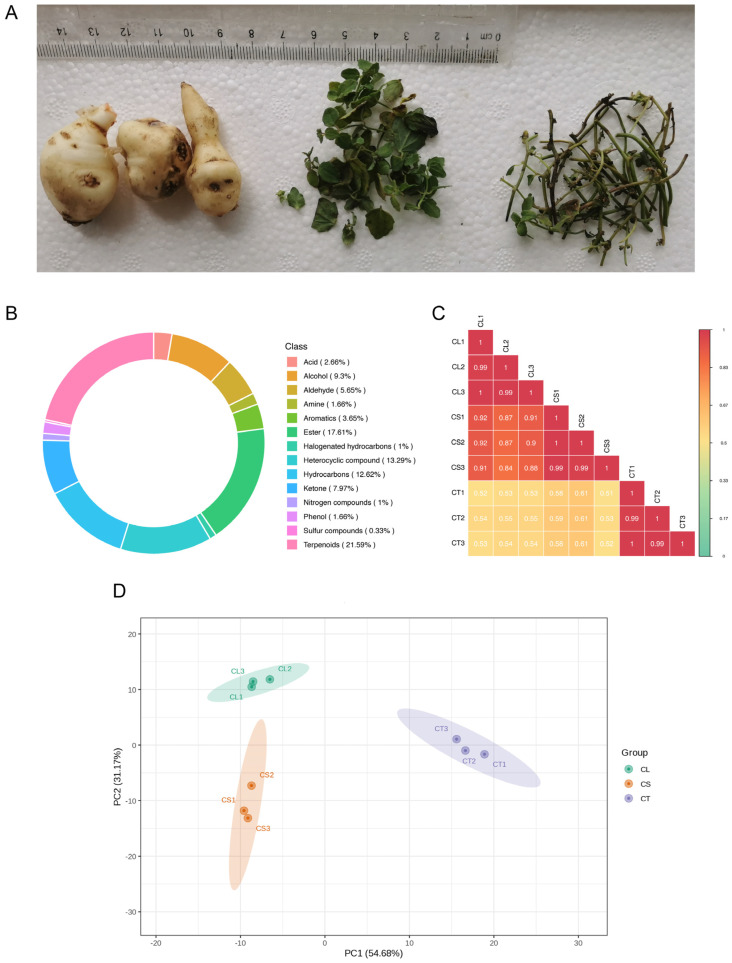
Metabolic characterization of leaf, stem, and tuber tissues of *C. convolvulacea*. (**A**) Pictorial representation of tuber, leaf, and stem samples (from left to right). (**B**) Identification of major metabolite classes detected. (**C**) Heatmap showing correlation of metabolite accumulation patterns between leaf (CL), stem (CS), and tuber (CT) tissues. Colors denote relative metabolite abundance. (**D**) Principal component analysis (PCA) depicting differences in the overall metabolite profile of the leaf (CL), stem (CS), and tuber (CT) samples.

In the comprehensive analysis of *C. convolvulacea*, a total of 302 unique volatile compounds were identified across various tissues, namely leaves, stems, and tubers (Figure 4A,B). After filtering the dataset for non-natural volatiles, 250 natural volatiles were retained for further analysis (Table S1). The dataset revealed the presence of 14 distinct classes of compounds, underscoring the plant’s diverse metabolic profile. Among these, the most predominant classes were terpenoids (60 occurrences), esters (34 occurrences), heterocyclic compounds (34 occurrences), hydrocarbons (35 occurrences), and alcohols (21 occurrences), indicating their significant presence in the plant’s tissues. Other notable classes included ketones, aldehydes, aromatics, acids, phenols, amino acids, nitrogen compounds, halogenated hydrocarbons, and sulfur compounds, albeit in smaller frequencies. This varied composition of volatile metabolites in *C. convolvulacea* highlights the intricate biochemical interactions within the plant and suggests a rich reservoir of phytochemicals that may be of interest for various applications in pharmacology and biochemistry. The analysis not only reflects the plant’s complex chemical ecology, but also opens up avenues for further research into the specific roles and potential of these compounds.

The correlation analysis of metabolite accumulation patterns across the different tissues of *C. convolvulacea* reveals interesting inter-relationships (Figure 4C). The correlation between leaf (CL) and stem (CS) tissues is notably high, with an average Pearson correlation coefficient of approximately 0.89. This strong correlation suggests a similar metabolite profile or coordinated metabolic activities between these two tissue types. In contrast, the correlation between leaf (CL) and tuber (CT) tissues and between stem (CS) and tuber (CT) tissues is comparatively lower, with average coefficients of about 0.54 and 0.57, respectively. These values indicate a less pronounced but still significant level of correlation, suggesting some commonalities in the metabolite accumulation patterns between these tissues, albeit to a lesser extent than between leaves and stems.

The principal component analysis (PCA) of the metabolite accumulation patterns in different tissues, as depicted in Figure 4D, demonstrates both the consistency of the data and the distinct metabolic profiles of the tissues. The replicated data within each tissue group (leaves, stems, and tubers) exhibited similar values, thereby affirming the reliability and quality of the experimental data. This consistency is evidenced by the clustering of replicates within each tissue group, indicating a high degree of reproducibility in the metabolite profiling. Furthermore, the PCA results clearly show that each group of tissues occupies a different region in the PCA plot (Figure 4D), highlighting significant differentiation in their metabolic profiles. The leaf, stem, and tuber tissues are distinctly separated in the PCA space, illustrating that each tissue type has a unique metabolic signature. This separation is indicative of the specialized metabolic processes and functions inherent to each tissue type in *C. convolvulacea*. Moreover, PC1 and PC2 covered a variation of 54.68% and 31.17%, respectively.

### 2.8. Differential Accumulation Pattern of Metabolites in Different Tissues of C. convolvulacea

**Figure 5:**
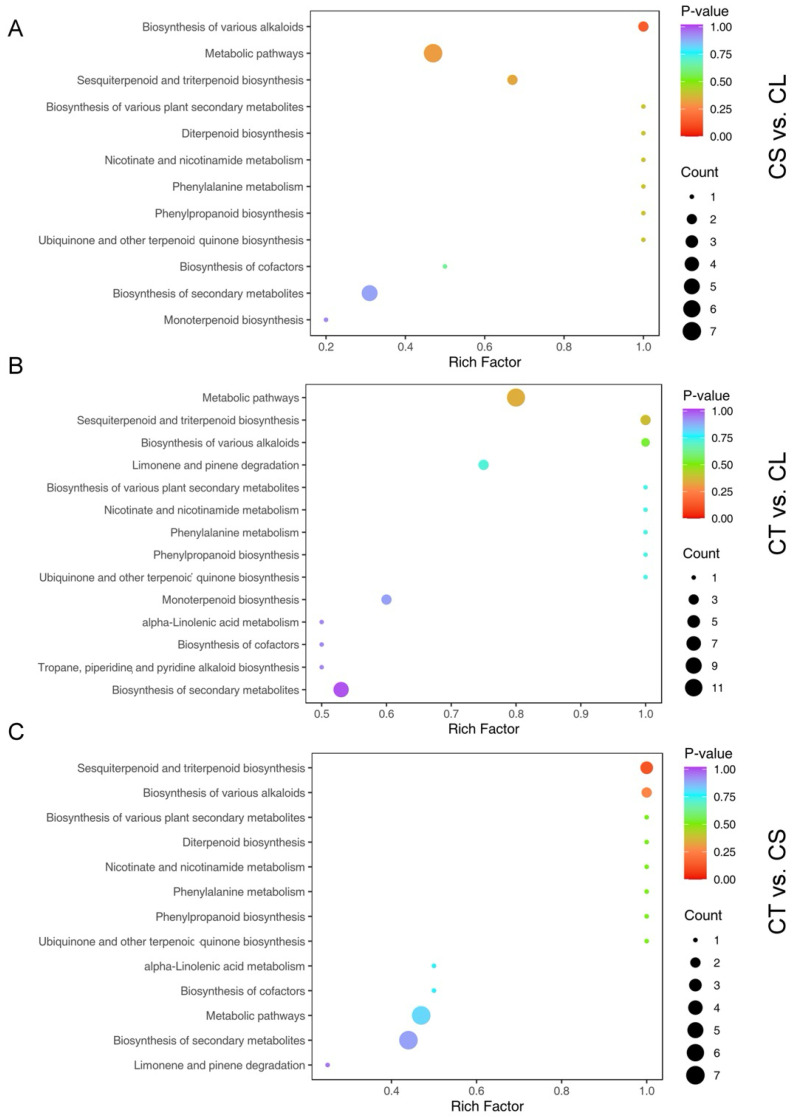
KEGG pathway enrichment analysis of differentially accumulated volatile metabolites: (**A**) stem (CS) vs. leaf (CL); (**B**) tuber (CT) vs. leaf (CL); (**C**) tuber (CT) vs. stem (CS). The analysis highlights pathways and processes particularly enriched in each tissue type, based on the metabolite profile.

In the comprehensive analysis of *C. convolvulacea*, the differential accumulation of volatiles across various tissues was distinctly evident. The study identified a significant number of differentially accumulated metabolites (DAMs) in pairwise comparisons among the three tissue types: leaves (CL), stems (CS), and tubers (CT). In the comparison between stem and leaf tissues (CS vs. CL), 115 significant DAMs were observed, with a predominant up-regulation in the stems as evidenced by 104 volatiles, compared to only 11 down-regulated volatiles. The tuber versus leaf comparison (CT vs. CL) revealed a more balanced distribution with 192 significant DAMs, comprising 97 up-regulated and 95 down-regulated in the tubers. In contrast, the tuber versus stem comparison (CT vs. CS) showed a distinct pattern with 159 DAMs, where 41 were up-regulated and 118 were down-regulated in the tubers. The KEGG enrichment analysis revealed significant enrichment of alkaloid biosynthesis and terpenoid biosynthesis in all three comparisons (Figure 5).

**Figure 6:**
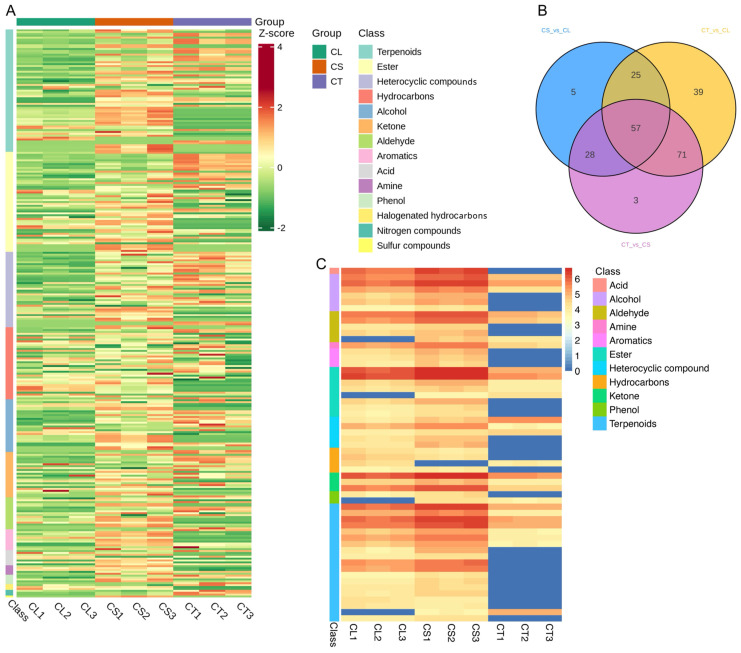
Metabolic profiling and comparisons of leaf, stem, and tuber tissues in *C. convolvulacea*. (**A**) Heatmap showing metabolite accumulation patterns across different subclasses in the leaf (CL), stem (CS), and tuber (CT). (**B**) Venn diagram illustrating overlaps in differentially accumulated volatile metabolites from pairwise comparisons of CT vs. CL, CT vs. CS, and CS vs. CL. (**C**) Heatmap depicting accumulation profiles of 57 common differentially accumulated volatile metabolites across the leaf (CL), stem (CS), and tuber (CT). The metabolic profiling revealed both shared and specialized metabolites across the three tissue types.

In the comprehensive analysis, a notable set of 57 volatiles was identified as being differentially accumulated across all three tissue types: leaves (CL), stems (CS), and tubers (CT) (Figure 6). These volatiles belong to a diverse range of chemical classes and exhibited unique accumulation patterns in each tissue comparison. Among them, certain classes were more prevalent, highlighting their potential significance in the plant’s metabolic processes. For example, terpenoids and esters were particularly prominent among these common differentially accumulated metabolites (DAMs). Compounds from the ester and terpenoid class were up-accumulated in the stem versus leaf comparison (CS vs. CL), and down-accumulated in both the tuber versus leaf (CT vs. CL) and tuber versus stem (CT vs. CS) comparisons. Niacinamide (an amino acid), another example from a different class, also followed this pattern of differential accumulation.

The prominence of classes such as terpenoids and esters among the 57 common volatile DAMs underscores their significant role in the diverse metabolic functions and adaptations specific to each tissue type in *C. convolvulacea* (Figure 6B,C). The identification of these volatiles, especially those belonging to the most prevalent classes, not only enriches our understanding of the plant’s biochemical diversity, but also suggests the intricate metabolic interactions and regulatory mechanisms within the plant. The consistent differential accumulation of these compounds across the tissue types may reflect their crucial role in the plant’s growth, development, and response to environmental factors.

The distinct metabolic profiles of the leaves (CL), stems (CS), and tubers (CT) were highlighted by the differential accumulation of specific volatile metabolites in each tissue type (Table S2). The analysis revealed the top ten volatiles that were predominantly accumulated in each of these tissues, underscoring the unique biochemical roles and functions they possess.

In the leaf tissues (CL), volatiles such as 8-Dodecen-1-ol, (Z)-, Tridecanal, p-Cymene, and 3-(4-Isopropylphenyl)-2-methylpropionAldehyde, featured prominently. These were accompanied by benzene, 1,2,4-trimethyl-, 1,5-Cyclodecadiene, 1,5-dimethyl-8-(1-methylethyl)-, 1-iodo-Decane, and 2-Buten-1-ol, 2-ethyl-4-(2,2,3-trimethyl-3-cyclopenten-1-yl)-. The list was rounded off by 3-MethoxycinnamAldehyde and [1aR-(1a.alpha.,4.alpha.,4a.beta.,7b.alpha.)]-1a,4,4a,5,6,7b-hexahydro-1,1,4a,7b-tetramethyl-1H-inden-4-ol. The accumulation of these metabolites in leaves suggests a specific biochemical role tailored to the leaf tissue’s functional requirements.

In the stem tissues (CS), a different set of metabolites were found to be predominant. These included 6-Octenoic Acid, 3,7-dimethyl-, 2-Tetradecene, (E)-, and bicyclo [3.2.1]oct-2-ene, 3-methyl-4-methylene-. Other significant metabolites were carveol, hexadecane, 4-methyl-, 1H-Pyrrole-2-carboxAldehyde, cyclohexanone, 5-methyl-2-(1-methylethylidene)-, and hexadecane, 2-methyl-. The accumulation patterns in stems reflect the distinct metabolic activities and stress response characteristics of stem tissue.

The tuber tissues (CT), crucial for storage and nutrient accumulation in *C. convolvulacea*, exhibited a unique profile with the presence of metabolites like 8-Dodecen-1-ol, (Z)-, Tridecanal, p-cymene, and 3-(4-Isopropylphenyl)-2-methylpropionAldehyde. Also included in the top 10 metabolites were Benzene, 1,2,4-trimethyl-, 1,5-Cyclodecadiene, 1,5-dimethyl-8-(1-methylethyl)-, 1-iodo-Decane, 2-Buten-1-ol, 2-ethyl-4-(2,2,3-trimethyl-3-cyclopenten-1-yl)-, 3-MethoxycinnamAldehyde, and [1aR-(1a.alpha.,4.alpha.,4a.beta.,7b.alpha.)]-1a,4,4a,5,6,7b-hexahydro-1,1,4a,7b-tetramethyl-1H-inden-4-ol. This distinct metabolite profile in tubers underscores their role in the plant’s growth and development.

### 2.9. Top Accumulated Volatiles in CS vs. CL

**Figure 7:**
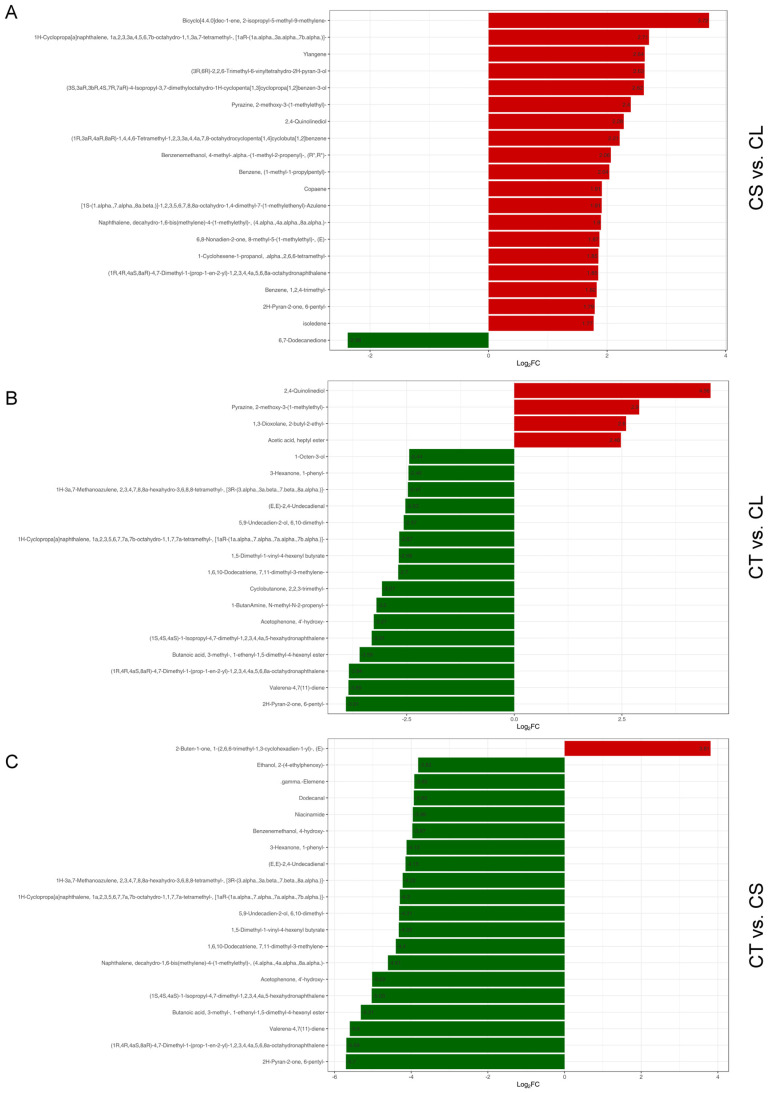
The top differentially accumulated volatile metabolites from pairwise tissue comparisons: (**A**) the top metabolites in the stem (CS) vs. leaf (CL); (**B**) the top metabolites in the tuber (CT) vs. leaf (CL); (**C**) the top metabolites in the tuber (CT) vs. stem (CS). The metabolites are ranked by the fold change in abundance between the compared tissues.

In the CS vs. CL comparative analysis, a discernible pattern of metabolite accumulation was observed, which is presented in Figure 7A. Among the metabolites with the highest fold changes, terpenoids were the most prominent class, indicative of their substantial role in the physiological variations between stem and leaf tissues. The list of the most positively accumulated terpenoids includes compounds such as Bicyclo[4.4.0]dec-1-ene, 2-isopropyl-5-methyl-9-methylene-, and 1H-Cyclopropa[a]naphthalene, a compound with a complex structure featuring multiple ring systems and methyl groups, suggesting potential roles in the plant’s defense mechanisms or growth processes. Ylangene, a fragrant compound known for its application in perfumery, also showed positive accumulation, possibly contributing to the olfactory characteristics of the stem tissue.

Other heterocyclic compounds, such as (3S,3aR,3bR,4S,7R,7aR)-4-Isopropyl-3,7-dimethyloctahydro-1H-cyclopenta [1,3] cyclopropa [1,2] benzen-3-ol, and Pyrazine, 2-methoxy-3-(1-methylethyl)-, were also up-accumulated in stem tissues. These compounds are known for their potential bioactivity, including antimicrobial properties. The presence of 2,4-Quinolinediol and other quinoline derivatives further underscores the stem tissue’s rich alkaloidal content, which can be crucial for pharmaceutical use, due to their broad spectrum of biological activity.

Benzenemethanol, 4-methyl-alpha, represents the alcohol class -(1-methyl-2-propenyl)-, an aromatic alcohol, which could play a role in the plant’s aroma profile or defense strategy. Notably, all the identified top differentially accumulated metabolites were positively accumulated in the CS vs. CL comparison. This particular ketone could be involved in different biochemical pathways or stress responses within the plant.

### 2.10. Top Accumulated Volatiles in CT vs. CL

In our comparative metabolic profiling, the analysis between tuber (CT) and leaf (CL) tissues revealed a distinctive pattern of differentially accumulated metabolites (Figure 7B). Among the top FC metabolites, heterocyclic compounds, such as 2,4-Quinolinediol, Pyrazine, 2-methoxy-3-(1-methyl ethyl)-, and 1,3-Dioxolane, 2-butyl-2-ethyl-, were found to be up-accumulated in tubers, suggesting an enhanced biosynthetic activity or a specific functional requirement for these metabolites in tuber tissues. In contrast, a diverse array of other metabolites, including the alcohol 1-Octen-3-ol, the ketone, and a selection of terpenoids, such as 1H-3a,7-Methanoazulene, were observed to be down-accumulated in tubers compared to leaves. This trend of down-accumulation also extended to compounds like 1,5-Dimethyl-1-vinyl-4-hexenyl butyrate, an ester, along with other terpenoids, which play pivotal roles in plant defense mechanisms and growth processes. Moreover, the presence of metabolites, such as Cyclobutanone, 2,2,3-trimethyl-, 1-ButanAmine, N-methyl-N-2-propenyl-, Acetophenone, 4′-hydroxy-, Valerena-4,7(11)-diene, and 2H-Pyran-2-one, 6-pentyl-, in lower concentrations in tubers, accentuates the complex metabolic interplay within the plant.

### 2.11. Top Accumulated Volatiles in CT vs. CS

In the CT vs. CS comparison (Figure 7C), our analysis revealed a diverse array of top FC metabolites, exhibiting differential accumulation patterns. Among these, the terpenoid 2-Buten-1-one, 1-(2,6,6-trimethyl-1,3-cyclohexadien-1-yl)-, (E)-, was the only metabolite to show an up-accumulation in tuber tissues compared to stems, suggesting an enhanced role or elevated biosynthesis in tuber tissue.

Conversely, a range of other metabolites among top FC metabolites displayed down-accumulation in tubers, including the alcohol Ethanol, 2-(4-ethylphenoxy)- and terpenoids, such as gamma-elemene and 1H-3a,7-Methanoazulene, highlighting a reduction in their concentrations or suppressed biosynthetic activity in CT compared to CS. The aldehydes, alongside the amine niacinamide and the ketone, also followed this trend, further emphasizing the distinct metabolic profiles of these tissues.

Other terpenoids, such as 1H-Cyclopropa[a]naphthalene and 1,6,10-Dodecatriene, continued this pattern of decreased levels in tuber tissues. The presence of alcohols, like 5,9-Undecadien-2-ol, and esters, such as 1,5-Dimethyl-1-vinyl-4-hexenyl butyrate, which were also down-accumulated, may reflect specific adaptations or metabolic needs unique to tuber tissues. Additionally, compounds like ketone Acetophenone, 4′-hydroxy-, and other terpenoid structures, were found to be less abundant in tubers, potentially indicating the tissue-specific regulation of metabolic pathways in *C. convolvulacea*.

## 3. Discussion

This comprehensive metabolomic study of *C. convolvulacea* provides valuable insights into the tissue-specific accumulation patterns of bioactive compounds in this important medicinal plant. The profiling of over 300 volatile metabolites across leaves, stems, and tubers, revealed both similarities and differences in their biochemical composition. Leaves and stems showed a high correlation in terms of their metabolite levels, likely reflecting their role in photosynthesis and aerobic respiration. In contrast, tubers displayed a more distinct profile, tailored to their specialized storage function.

Leaves were characterized by high levels of terpenoids, ester, and alcohol. This aligns with their photosynthetic activity and production of photoprotective pigments and compounds [16,17,18]. The abundance of bioactive compounds gives leaves potential medicinal utility [19]. Stems accumulated more terpenoids, heterocyclics, and alkaloids than leaves. Many terpenoids have antimicrobial, anti-inflammatory, and anticancer properties, suggesting stems may be useful pharmaceutically [20,21,22,23]. Tubers were enriched in carbohydrates like sugars and starch, befitting their storage function. However, they still contained reasonable levels of terpenoids, alkaloids, and other bioactive metabolites. This indicates tubers can provide nutritional and medicinal value, despite being weaker than leaves or stems in this regard.

Notably, terpenoids and esters featured prominently as differentially accumulated metabolites (DAMs) in all tissue comparisons. Their consistent accumulation underscores their key roles in growth, development, and stress adaptation [24,25,26]. Other classes like heterocyclics, aldehydes, and alcohols also exhibited tissue-preferential accumulation, highlighting the complex, compartmentalized biochemistry within the plant.

The identification of tissue-specific metabolic signatures provides a rational basis for selective utilization of *C. convolvulacea* parts. Leaves could be valuable for antioxidants, stems for drug development, and tubers for traditional tonics or nutraceuticals. Targeted extraction and processing methods can be devised to optimally harness the tissue-specific phytochemical reservoirs. For instance, we identified that niacinamide was significantly accumulated in leaves compared to stems and tubers. Niacinamide, also known as nicotinamide, is the amide form of niacin or vitamin B3 [27]. As a hydrophilic vitamin derivative, niacinamide exhibits diverse pharmacological effects on cutaneous physiology [28]. In recent years, niacinamide has gained considerable recognition as a versatile cosmeceutical agent and is now extensively incorporated into topical skincare formulations [29,30,31,32]. The epidermal benefits of niacinamide stem from its capacity to rebuild keratin, stimulate ceramide and protein synthesis, exhibit anti-inflammatory properties, improve epidermal barrier function, regulate pigmentation, and suppress melanosome transfer [33].

Another significantly up-accumulated compound in leave tissue was tridecanal, which is a composite of essential oil extracted from several other plant species, including but not limited to *Styrax officinalis* [34], *Cnidium officinale* [35], *Coriandrum sativum* [36], and *Litsea fulva* [37]. Essential oils extracted from plants have become indispensable raw materials in various industries, including food, beverage, fragrance, pharmaceutical, and cosmetic sectors [38]. As natural plant extracts, essential oils impart flavor and aroma to many consumable goods [39,40,41]. Additionally, some essential oils exhibit bioactive properties that make them useful for health and beauty products [41]. Moreover, stem tissues showed significant accumulation of benzeneacetic acid and carveol. Benzeneacetic acid has been used as a preservative in the cosmetics industry [42], and as a flavoring agent in food, cosmetic, and pharmaceutical products [43]. While carveol is a monoterpene alcohol naturally found in the essential oils of certain plants [44]. It has been identified as a constituent in essential oils extracted from *Cymbopogon giganteus* [45], *Illicium pachyphyllum* [46], and the spice *Carum carvi* (cumin) [47]. Emerging research indicates that carveol demonstrates bioactive properties that may have pharmaceutical applications. Specifically, in vitro and animal studies have revealed antitumor, antimicrobial, neuroprotective, vasorelaxant, antioxidant, and anti-inflammatory activities associated with carveol [48,49,50]. In addition, p-cymene is a monoterpene compound that has been identified as a component of the metabolites from the tubers of *C. convolvulacea*. As an aromatic hydrocarbon, p-cymene is found widely distributed across the plant kingdom and contributes to the essential oils of numerous species [51,52]. Beyond its natural occurrence, p-cymene has attained significance as a versatile industrial precursor [51]. It is employed synthetically in the production of pesticides, fungicides, pharmaceuticals, antioxidants, and an array of fragrance ingredients for cosmetics and consumer goods [53,54,55,56]. The discovery of p-cymene, among the phytochemical constituents of *C. convolvulacea,* provides further evidence of this plant’s potential multipurpose applications stemming from its rich metabolic diversity.

The comprehensive metabolomic analysis of volatiles in *C. convolvulacea* across three tissue types has identified a unique set of top FC volatiles for each tissue comparison, underscoring their distinct biochemical profiles. In the CS vs. CL comparison, terpenoids were notably up-accumulated in stem tissues, suggesting a potential for valorization in phytopharmaceuticals due to their bioactive properties. Conversely, in the CT vs. CL and CT vs. CS comparisons, a variety of metabolites, including terpenoids, alcohols, and ketones, were predominantly down-accumulated in tuber tissues, indicating more reserved metabolic activity in these parts.

## 4. Conclusions

In summary, *C. convolvulacea* leaves, being rich in specific aromatic compounds, could be explored for their antioxidative and antimicrobial potential, while the stems, with their abundance of terpenoids, may hold promise for applications in anti-inflammatory and anticancer drug development. Tubers, displaying a diverse metabolic composition with a significant presence of bioactive compounds, despite some down-accumulation, could be investigated for their use in nutraceuticals and traditional medicine formulations. These findings pave the way for targeted valorization strategies, exploiting the differential metabolite accumulation in various tissues of *C. convolvulacea* for medicinal and therapeutic applications.

Furthermore, this knowledge can inform selective breeding strategies to enhance bioactive compound yields in different plant parts. The overexpression of relevant biosynthetic genes in target tissues through transgenic approaches may boost yields. Identifying genetic loci regulating tissue-specific accumulation can facilitate marker-assisted selection.

## 5. Materials and Methods

### 5.1. Plant Material and Sample Collection

Healthy, consistently growing *Codonopsis convolvulacea* Kurz var. vinciflora plants were selected and harvested in September 2021 from a natural habitat in Nyingchi, Tibet Autonomous Region, China (latitude 29°40′23.40″ N, longitude 94°20′28.28″ E, elevation 3000 m). Three key tissues, namely tubers, leaves, and stems, were collected from multiple plants at the same developmental stage. Only young, fully expanded leaves were taken. Stems were cut into 5 cm segments, avoiding woody basal parts. Intact tubers of comparable size were uprooted. The collected tissues were thoroughly washed with tap water to remove soil particles, followed by rinsing three times with double-distilled water to eliminate surface contaminants. The washed tissues were gently patted dry with paper towels to absorb excess moisture. Approximately 100 g of each tissue type was immediately frozen in liquid nitrogen in the field, placed in labeled cryovials, and stored at −80 °C until metabolite extraction.

### 5.2. Metabolite Extraction

The samples were pulverized into a fine powder under liquid nitrogen conditions. Approximately 500 mg (1 mL) of the powdered sample was then placed into a 20 mL headspace vial (sourced from Agilent, Palo Alto, CA, USA), which contained a sodium chloride saturated solution to prevent any enzymatic activity. These vials were securely sealed with crimp-top caps, fitted with TFE–silicone headspace septa (provided by Agilent). Prior to the solid-phase microextraction (SPME) analysis, each vial was warmed to 60 °C for five minutes. Subsequently, a 120 µm DVB/CWR/PDMS fiber (also from Agilent) was introduced into the vial’s headspace for 15 min, at the same temperature, to absorb volatile organic compounds (VOCs).

### 5.3. Metabolite Profiling

For gas chromatography–mass spectrometry (GC-MS) conditions, the volatile compounds (VOCs) were thermally desorbed from the fiber coating within the injection port of the GC system (Model 7890B; Agilent) at 250 °C for five minutes, under splitless conditions. The VOCs were then identified and quantified using a GC system paired with a 7000D mass spectrometer (Agilent), which utilized a 30 m × 0.25 mm × 0.25 μm DB-5MS capillary column coated with 5% phenyl-polymethylsiloxane. Helium served as the carrier gas, flowing at a constant rate of 1.2 mL/min. The temperatures for the injector and detector were maintained at 250 °C and 280 °C, respectively. The oven’s temperature was initially held at 40 °C for 3.5 min, then ramped up by 10 °C/min to 100 °C, followed by an increase of 7 °C/min to 180 °C, and finally by 25 °C/min to a maximum of 280 °C, where it was held for an additional five minutes. The mass spectra were acquired in electron impact (EI) ionization mode at 70 eV, with the quadrupole mass detector, ion source, and transfer line temperatures set at 150, 230, and 280 °C, respectively. For the analyte identification and quantification, the mass spectrometer was operated in selected ion monitoring (SIM) mode.

To identify differentially accumulated metabolites, statistical analyses were performed using the fold change (FC), *p*-value, and variable importance in projection (VIP) score. Metabolites with a fold change greater than 2 or less than 0.5 between the experimental groups were considered significant. The fold change was calculated as the ratio of metabolite abundance in the treatment group to the control group. Student’s *t*-test was conducted to determine the statistical significance of the metabolite differences between the groups. A p-value less than 0.05 was considered statistically significant. Multiple testing correction was applied using the Benjamini–Hochberg method to control the false discovery rate (FDR). For multivariate analysis, partial least squares discriminant analysis (PLS-DA) was employed. Metabolites with VIP scores greater than 1.0 were selected as potential biomarkers, due to their high contribution to the model’s classification performance.

### 5.4. Antioxidant Activity Assessment

The antioxidant activity of different plant parts (leaves, stems, and tubers) was assessed using three different assays: a DPPH radical scavenging assay, an ABTS radical cation decolorization assay, and a ferric reducing antioxidant power (FRAP) assay. The samples were homogenized and prepared as described below.

Plant tissues were collected, immediately frozen in liquid nitrogen, and stored at −80 °C until further analysis. Prior to the assays, the samples were thawed and homogenized using a blender to ensure uniformity. Approximately 1 g of each homogenized sample was extracted with 10 mL of 80% methanol by vortexing for 10 min. The extracts were centrifuged at 12,000 rpm for 10 min at 4 °C, and the supernatant was collected for antioxidant activity assays.

The DPPH radical scavenging activity was determined using a modified method based on Brand-Williams et al. (1995). A 0.1 mM solution of DPPH in methanol was prepared, and 2 mL of this solution was mixed with 2 mL of the sample extract. The mixture was incubated in the dark at room temperature for 30 min. The absorbance was measured at 517 nm using a spectrophotometer. The DPPH radical scavenging activity was calculated using the formula:

DPPH Activity (U/g)=(Acontrol−Asample)/Acontrol)×100

where A*_control_* is the absorbance of the control (DPPH solution without sample), and A*_sample_* is the absorbance of the sample extract.

The ABTS radical cation decolorization assay was performed according to Re et al. [57], with slight modifications. The ABTS radical cation was generated by mixing 7 mM ABTS solution with 2.45 mM potassium persulfate and allowing the mixture to stand in the dark at room temperature for 12–16 h. The ABTS solution was then diluted with ethanol to an absorbance of 0.70 ± 0.02 at 734 nm. A 2 mL aliquot of the ABTS solution was mixed with 20 μL of sample extract, and the absorbance was measured at 734 nm after 6 min. The ABTS radical scavenging activity was calculated as follows:

ABTS activity (U/g)=(Acontrol−Asample)/Acontrol)×100

where A*_control_* is the absorbance of the control (ABTS solution without sample), and A*_sample_* is the absorbance of the sample extract.

The FRAP assay was conducted based on the method described by Benzie and Strain [58]. The FRAP reagent was prepared by mixing 300 mM acetate buffer (pH 3.6), 10 mM TPTZ solution in 40 mM HCl, and 20 mM FeCl₃·6H₂O in a ratio of 10:1:1 (*v*/*v*/*v*). A 3 mL aliquot of the FRAP reagent was mixed with 100 μL of sample extract and incubated at 37 °C for 4 min. The absorbance was measured at 593 nm. The antioxidant power was expressed as μmol Fe(II) equivalents per gram of sample (μmol/g), calculated from a standard curve generated with FeSO₄·7H₂O.

### 5.5. Determination of Proximate Composition

The crude protein, crude fat, ash, and moisture content were analyzed using standard AOAC methods. The nitrogen content was measured by the Kjeldahl method, and the crude protein was calculated using a conversion factor of 6.25. Approximately 2 g of the dried, ground sample was digested with concentrated sulfuric acid and a catalyst, until the mixture was clear. The digest was neutralized with sodium hydroxide and distilled to release ammonia, which was then trapped in a boric acid solution and titrated with standard hydrochloric acid.

For the crude fat quantification, approximately 5 g of the dried, ground sample was placed in a Soxhlet extractor. The sample was extracted with petroleum ether for 4–6 h. The solvent was then evaporated, and the residue was dried in an oven at 70 °C until a constant weight was achieved, before being weighed gravimetrically.

The ash content was determined by placing approximately 2 g of the sample into a crucible and incinerating it in a muffle furnace at 550 °C until a constant weight was achieved. The moisture content was measured in triplicate by weighing about 5 g of the sample and drying it in an oven at 70 °C until the weight was constant. All proximate analyses were performed at the laboratories of Norminkoda Biotechnology Co., Ltd. (Wuhan, China).

### 5.6. Determination of Amino Acids

The total amino acid content was quantified by the ninhydrin colorimetric method, using a biochemical kit (NMKD0262, Norminkoda). Approximately 200 mg of the homogenized sample was weighed and hydrolyzed with 6M HCl at 110 °C for 22 h in a sealed tube. After hydrolysis, the solution was filtered and evaporated to dryness under reduced pressure, and the residue was dissolved in a suitable buffer for the ninhydrin reaction. The amino acids reacted with the ninhydrin reagent to form a purple complex, which was measured spectrophotometrically at 570 nm. Glycine was used to generate a standard curve, and the results expressed as g/100 g glycine equivalents.

### 5.7. Determination of Total Alkaloids

The total alkaloid content was determined by a spectrophotometric method, using bromocresol green. Alkaloids form a yellow-colored complex, with bromocresol green showing maximum absorbance at 415 nm. About 1 g of the homogenized sample was mixed with 10 mL of acidified water and allowed to stand for 2 h. The mixture was filtered, and the filtrate was made alkaline with ammonia, before being extracted with chloroform. The extract was evaporated to dryness, and the residue was dissolved in bromocresol green reagent. The alkaloids formed a yellow-colored complex with bromocresol green, showing maximum absorbance at 415 nm. Absorbance was measured at 415 nm, and the total alkaloids were quantified based on a calycanthine standard curve, with the results reported as g/100 g calycanthine equivalents.

### 5.8. Determination of Total Sugars

The total sugar content was measured by the anthrone–sulfuric acid method, using a biochemical kit (NMKD0206, Norminkoda). Sugars react with anthrone reagents under heated acidic conditions to produce a green-colored complex. The absorbance was read at 620 nm, and the total sugars were calculated from a glucose standard curve and expressed as g/100 g glucose equivalents.

### 5.9. Determination of Starch Content

The starch content was determined using an enzymatic assay kit (NMKD0213, Norminkoda). Starch was converted to glucose by the amyloglucosidase enzyme. The released glucose was then oxidized by glucose oxidase, and the resulting hydrogen peroxide reacted with a chromogenic dye to generate color. The absorbance was measured at 505 nm, and the starch content was calculated from a starch standard curve and reported as g/100 g starch equivalents.

### 5.10. Statistical Analysis

The data obtained from the phytochemical analyses of the leaves, stems, and tubers were statistically analyzed using GraphPad Prism 9.3.1 software. The results were expressed as mean ± standard deviation (SD) of three independent measurements. The differences between the tissue types were assessed by paired two-tailed *t*-tests. Moreover, *p* values < 0.05 were considered statistically significant.

For each phytochemical constituent assayed, including total phenolics, flavonoids, alkaloids, amino acids, sugars, and starch, the mean concentration from each tissue (from the leaf, stem, tuber) was compared in a pairwise fashion. Paired *t*-tests were applied to compare leaves vs. stems, leaves vs. tubers, and stems vs. tubers, for each constituent. Statistically significant differences between the tissue pairs were identified based on *p*-values < 0.05. The paired *t*-test analysis enabled statistical determination of the differential accumulation of various phytochemicals among the assayed plant parts.

## Data Availability

All data used in this work can be found in the text and in the Supplementary Tables.

## Supplementary Materials

The following supporting information can be downloaded at: https://www.mdpi.com/article/10.3390/molecules29133193/s1. Table S1. Identification and characterization of volatiles in leaf (CL), stem (CS), and tuber (CT) tissues of *C. convolvulacea.* Table S2. Differential metabolic accumulation in leaf (CL), stem (CS), and tuber (CT) tissues of *C. convolvulacea.*
